# The impact of the atrial wall thickness in normal/mild late‐gadolinium enhancement areas on atrial fibrillation rotors in persistent atrial fibrillation patients

**DOI:** 10.1002/joa3.12676

**Published:** 2022-01-13

**Authors:** Toshihiro Nakamura, Kunihiko Kiuchi, Koji Fukuzawa, Mitsuru Takami, Yoshiaki Watanabe, Yu Izawa, Makoto Takemoto, Jun Sakai, Atsusuke Yatomi, Yusuke Sonoda, Hiroyuki Takahara, Kazutaka Nakasone, Kyoko Yamamoto, Yuya Suzuki, Ken‐ichi Tani, Noriyuki Negi, Atsushi Kono, Takashi Ashihara, Ken‐ichi Hirata

**Affiliations:** ^1^ Section of Arrhythmia Division of Cardiovascular Medicine Department of Internal Medicine Kobe University Graduate School of Medicine Kobe Japan; ^2^ Department of Radiology Kobe University Graduate School of Medicine Kobe Japan; ^3^ Division of Radiology Center for Radiology and Radiation Oncology Kobe University Hospital Kobe Japan; ^4^ 13051 Department of Medical Informatics and Biomedical Engineering Shiga University of Medical Science Otsu Japan

**Keywords:** atrial fibrillation, atrial wall thickness, fibrosis, late‐gadolinium enhancement magnetic resonance imaging, rotor

## Abstract

**Background:**

Some of atrial fibrillation (AF) drivers are found in normal/mild late‐gadolinium enhancement (LGE) areas, as well as moderate ones. The atrial wall thickness (AWT) has been reported to be important as a possible AF substrate. However, the AWT and degree of LGEs as an AF substrate has not been fully validated in humans.

**Objective:**

The purpose of this study was to evaluate the impact of the AWT in normal/mild LGE areas on AF drivers.

**Methods:**

A total of 287 segments in 15 persistent AF patients were assessed. AF drivers were defined as non‐passively activated areas (NPAs), where rotational activation was frequently observed, and were detected by the novel real‐time phase mapping (ExTRa Mapping), mild LGE areas were defined as areas with a volume ratio of the enhancement voxel of 0% to <10%. The AWT was defined as the minimum distance from the manually determined endocardium to the epicardial border on the LGE‐MRI.

**Results:**

NPAs were found in 20 (18.0%) of 131 normal/mild LGE areas where AWT was significantly thicker than that in the passively activated areas (PAs) (2.5 ± 0.3 vs. 2.2 ± 0.3 mm, *p* < .001). However, NPAs were found in 41 (26.3%) of 156 moderate LGE areas where AWT was thinner than that of PAs (2.1 ± 0.2 mm vs. 2.23 ± 0.3 mm, *p* = .02). An ROC curve analysis yielded an optimal cutoff value of 2.2 mm for predicting the presence of an NPA in normal/mild LGE areas.

**Conclusion:**

The location of AF drivers in normal/mild LGE areas might be more accurately identified by evaluating AWT.

## Graphical abstract

This study evaluated that the impact of the AWT in normal/mild LGE areas on AF rotors. In this study, NPAs were found in 20 (15.3%) of 131 normal/mild LGE areas where the AWT was significantly thicker in the NPAs than PAs. However, NPAs were found in 41 (26.3%) of 156 moderate LGE areas where the AWT was thinner than that of the PAs. AF, atrial fibrillation; AWT, atrial wall thickness; LGE, late gadolinium enhancement; NPA, non‐passively activated area; PA, passively activated area.

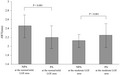

## 1 INTRODUCTION

Pulmonary vein isolation (PVI) is a well‐established ablation strategy for paroxysmal atrial fibrillation (AF), but it is much less effective in persistent AF patients.^1^ Late‐gadolinium enhanced magnetic resonance imaging (LGE‐MRI) has been reported to detect myocardial fibrosis. Furthermore, the progression of atrial fibrosis after catheter ablation may be associated with AF recurrence.^2^ It has been previously reported that AF rotors are observed in patchy LGE areas but not in dense LGE areas, in computer simulation models.^3^ That shows the importance of a qualitative and quantitative analysis of the LGE areas. Recently, the modulation of the AF rotors has been proposed as one of the effective ablation strategies for persistent AF.^4^ To evaluate the location of AF rotors precisely, a novel phase‐mapping system (ExTRa Mapping^TM^; Nihon Kohden) has been developed. It has been shown that reducing the number of rotors detected by ExTRa Mapping leads to a reduction in AF maintenance.^5^ ExTRa Mapping is a phase map based on myocardial action potentials, which has been validated by high‐resolution optical membrane potential mapping in an animal study. ExTRa Mapping has some reliability for analyzing the activation pattern the region of interest.^6^ We previously reported that AF rotors detected by the ExTRa Mapping were frequently found in moderate LGE areas assessed by LGE‐MRI in persistent AF patients. However, some of them were also found in normal/mild LGE areas.^7^ This has implied that there are other possible structural factors associated with AF rotors. A previous computer simulation study demonstrated the role of the atrial wall thickness (AWT) as a substrate for AF rotors and marker for the identification of AF rotor locations in patient‐specific atria, and the AWT gradients acted as anchoring points for AF rotors in the absence of fibrosis.^8^ However, such an effect of the AWT on AF rotors has not been fully verified in humans. The aim of this study was to evaluate the impact of the AWT in normal/mild LGE areas on AF rotors in persistent AF patients.

## 2 METHODS

### 2.1 Study population

A total of 15 consecutive patients with persistent (*n* = 6) and long‐standing persistent (*n* = 9) AF undergoing catheter ablation were enrolled in this study. The protocol of this research project has been approved by the appropriately constituted ethics committee of the institution concerned and complies with the provisions of the Declaration of Helsinki, Committee of 2021.5.25, Approval No. 210043.

### 2.2 MRI acquisition

Before the AF ablation, LGE‐MRI was performed in all patients using a 1.5T MR system (Achieva; Philips Medical) equipped with a five‐channel cardiac coil. This scan technique has been previously reported.^9^ First, contrast‐enhancement magnetic resonance angiography (CE‐MRA) of the pulmonary vein (PV)‐left atrial (LA) anatomy was obtained in the coronal plane using a breath‐hold three‐dimensional (3D) fast field echo sequence after the injection of 0.1 mmol/kg of a contrast agent (Gadobutrol, Gadovist, Bayer Yakuhin).^10^ The purpose of the scanning in the coronal plane was to reduce the number of acquisition slices and shorten the breath‐hold time. Next, 15 min after the contrast injection, LGE‐MRI of the LA including the PVs was performed using a lateral 3D inversion recovery, respiratory navigation, ECG gating, and T1‐fast field echo sequence.^11^ The CE‐MRA and LGE‐MRI images were transferred to customized software (MRI LADE Analysis; PixSpace Inc) for image post‐processing and an image analysis.

### 2.3 3D Visualization and assessment of the tissue properties

To detect normal/mild LGE areas more sensitively, we used the same protocol as in our previous study.^12^ The 3D visualization method for the LGE was as follows. First, the LA in the LGE‐MRI was semi‐manually segmented by contouring the borders between the endocardium and epicardium of the atrium, including the PVs, with reference to the CE‐MRA. Second, the mean value and standard deviation (SD) of the voxel intensity was measured on the “healthy” LA wall where no hyper‐enhanced areas in LGE‐MRA were involved. Third, we identified LGEs with an intensity of >1 SD on the "healthy" LA wall by a voxel intensity histogram analysis of the LA wall. Furthermore, the degree of the intensity was categorized by a color‐coded scaling (green: >1 SD: yellow: 2–3 SD; red: >3 SD). Finally, the 3D reconstruction, color‐coded LGE, and volume‐rendered LA and PV image generated from the CE‐MRA were semi‐automatically fused. In this study, atrial fibrosis was defined as an LGE site with a signal intensity of >1 SD. To evaluate the fibrotic tissue properties, the fibrotic density was measured as the LGE‐volume. The fibrotic density was defined as the volume ratio of an LGE signal intensity >1 SD (LGE‐volume ratio). The details of the measurement can be found in the previous publication.^7^ In this study, the areas with an LGE‐volume ratio of 0% were defined as normal areas, the areas with an LGE‐volume ratio of 0%–10% were defined as mild LGE areas, and moderate LGE areas were defined as areas with an LGE‐volume ratio of >10%.

### 2.4 Thickness measurement of the LA

**FIGURE 1:**
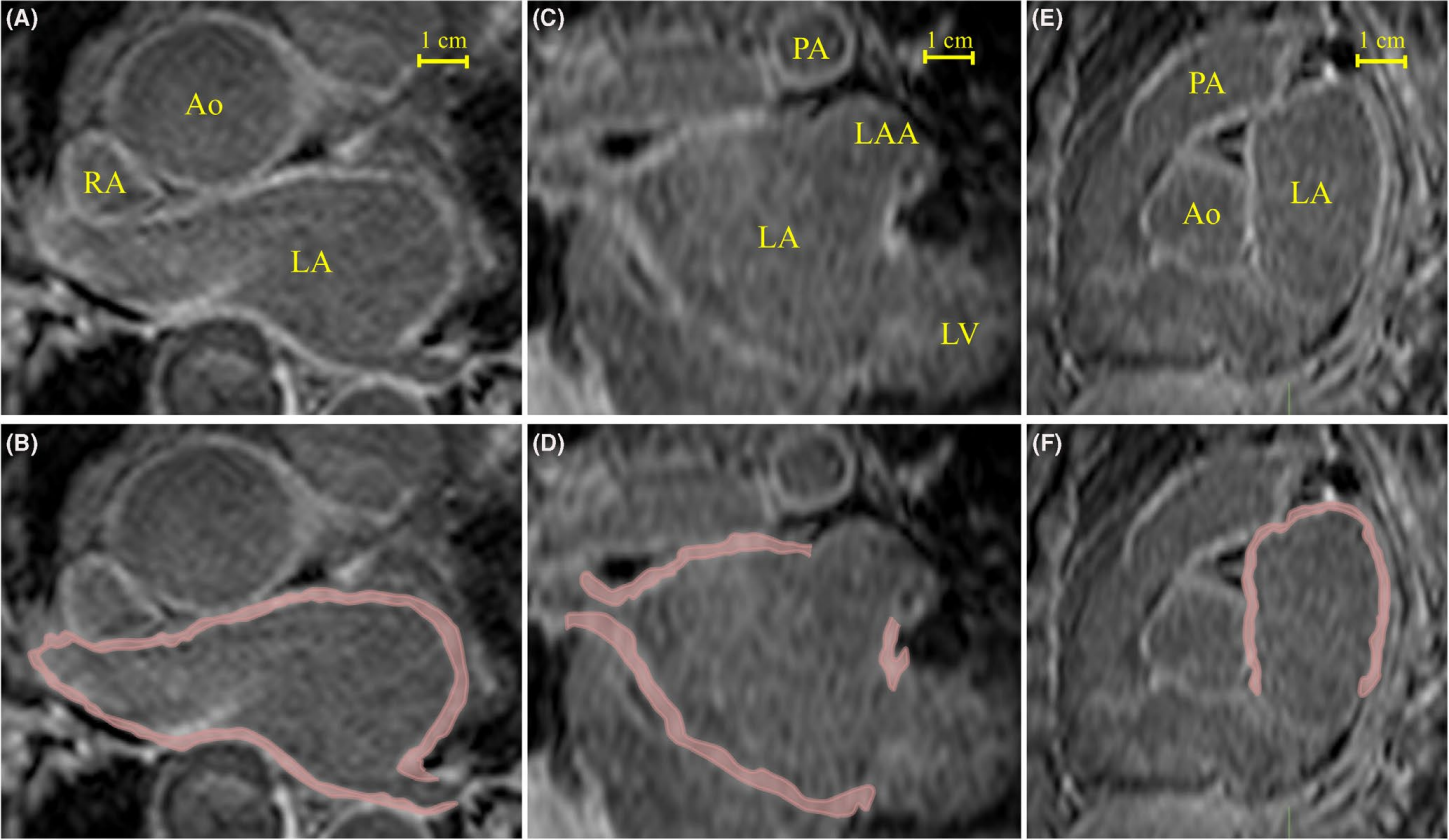
The measurement of the atrial wall thickness in our representative case. (A, B) Axial, (C, D) coronal and (E, F) sagittal views of the atria from one representative subject overlaid, in the bottom row, with the performed manual segmentations. Ao, aorta; LA, left atrium; LAA, left atrial appendage; LV, left ventricle; PA, pulmonary artery; RA, right atrium

As shown in Figure 1, the atrial wall thickness (AWT) was defined as the minimum distance from the manually determined endocardium to the epicardial border on the LGE‐MRI. Regions of interest were manually drawn in specific atrial regions and the regions of interest‐based AWT was estimated in the multiplanar reconstruction images perpendicular to the LA wall. To evaluate the AWT in the normal/mild LGE areas associated with AF rotors, a receiver operating characteristic (ROC) curve analysis was performed for the optimal values of the AWT predicting AF rotors.

### 2.5 Real‐time phase mapping

**FIGURE 2:**
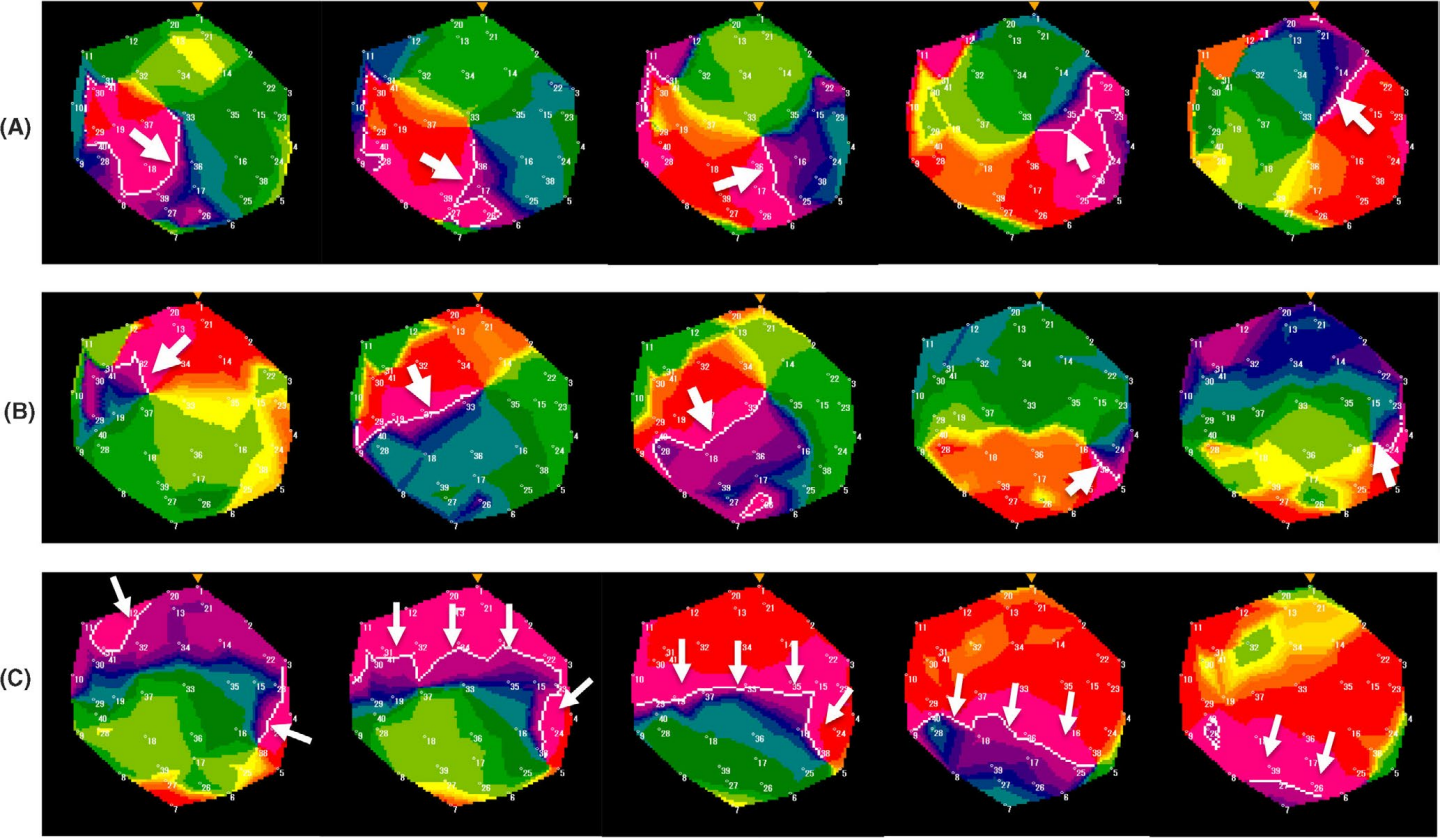
An example of ExTRa Mapping. White lines indicate the head of the wavefronts, and white arrows indicate the direction of the wavefronts. The panel (A) and (B) show the representative case of non‐passive activation where counterclockwise rotational activations with meandering the center of the activation was found. The panel (C) shows the representative case of passive activation where planar wave is found

After the integration of the anatomical 3D models of the LA and PVs obtained from the MRI, mapping was performed using the NavX system (Abbott) as a guide. A 20‐pole circular mapping catheter (Optima^TM^ or Reflexion HD^TM^, Abbott) and ablation catheter‐reconstructed LA posterior anatomy was aligned with the MRI.^13^ To detect the distribution of the AF rotors, an online real‐time phase mapping system (ExTRa Mapping) was used. The detail of this mapping system was previously described.^7^ We evaluated all areas where the mapping catheter has reached. ExTRa Mapping was applied to persistent AF patients and as a result, each wave dynamics were classified into three patterns, meandering rotors (MRs), multiple wavelets (MWs), and planar wave. Planar wave propagation was defined as passive activation, whereas MR and MW were defined as non‐passive activations. We described the example of ExTRa Mapping in Figure 2. Moreover, ExTRa Mapping system provides a "reliability signal" to monitor the quality of the signal. The reliability signals were colored as blue, green, orange, yellow, red, or gray according to the number of electrode pairs with impaired signal. Signals were recorded when a "reliability signal" was colored by blue, green, orange. When mapping catheter could not be adjusted, the signal sensing threshold was changed from 0.03 to 0.01 mV. Furthermore, non‐passively activated areas (NPAs), a region where non‐passive activations were frequently observed, were automatically detected according to the value of the “non‐passively activated ratio (%NP)” (the ratio of the form of MRs and/or MWs assumed to contain AF rotors to the recording time).^5^ NPAs were determined as areas up to the top 7 highest %NP values greater than 50%, corresponding to 1/4 to 1/3 surface area of the whole LA. Thus, the NPAs could be considered as the area where AF rotors could be frequently found. Pseudo‐rotors are not the main limitation because the phase map is reconstructed based on transmembrane voltage phase. To evaluate the distribution of the NPAs, the region of the whole LA was divided into the following 8 segments: PV antrum, roof, anterior, posterior, lateral, bottom, septum, and left atrial appendage (LAA) base segments. Moreover, we evaluated the proportion of MRs and MWs in the %NP within the NPAs in the normal/mild and moderate LGE areas.

### 2.6 Relationship between the AWT in the normal/mild LGE areas and AF rotors

To clarify the relationship between the AWT in the normal/mild LGE areas and AF rotors, the following were assessed: (1) the distribution of the NPAs, (2) correlation between the AWT and LGE‐volume ratio in the NPAs, and (3) optimal AWT in the normal/mild LGE areas for predicting the NPAs.

### 2.7 Ablation strategy

Basically, the aim of our study was to evaluate the relationship between the AWT and AF rotors on the normal/mild LGE areas but not the impact on the NPA ablation. Concerning the ablation strategy, if an NPA was found at the PV antrum, we attempted to create a PVI line that included the NPAs. If an NPA was found on the LA posterior wall, we added a Box lesion including the NPAs. If an NPA was found at other sites such as on the LA septum or near the left atrial appendage, those NPAs were left alone. After proving bidirectional block of the PVs or Box lesion, a stimulation protocol (burst pacing from the coronary sinus at 300, 250, and 200 ms for 10 s each) was performed to test the macro‐reentrant atrial tachycardia inducibility. When AF was induced, the patients were cardioverted and the procedure was ended. No pharmacological identification of non‐PV triggers was performed. Ablation of the cavotricuspid isthmus was performed only if a typical right atrial flutter was either documented previously or induced by burst pacing at the end of the procedure. Additional linear ablation was also added when the other macro‐reentrant tachycardia was induced.

### 2.8 Statistical analysis

Data are expressed as percentages for the nominal variables, medians for the ordinal variables, and means for the continuous variables. Discrete variables were compared using the chi‐square or Fisher exact test as appropriate. The mean AWT was compared among the eight segments of the whole LA groups using a one‐way ANOVA and post hoc analysis with a Tukey correction for multiple comparisons of data. ROC curves were used to determine the AWT that provided the best sensitivity and specificity for the NPAs. A value of *p* < .05 was considered statistically significant. The correlations between two parameters were assessed using Pearson or Spearman rank correlation tests. To assess the proportion of NPAs in each group, a correction for multiple comparisons was performed. All statistical analyses were performed using EZR on R commander, version 1.36 software.

## 3 RESULTS

### 3.1 Patient and procedural characteristics

The patient and procedural characteristics are shown in Table 1. The mean age was 66 ± 12 years, mean left atrial dimension 43 ± 8 mm, and mean left ventricular ejection fraction 60 ± 8%. Ten (67%) out of 15 patients underwent an initial AF catheter ablation. The time from the MRI acquisition to the AF ablation was 95 ± 60 days. The mean AWT in 287 areas in the LA in 15 patients was 2.2 ± 0.3 mm.

**TABLE 1:** Patients and procedural characteristics

	Total (*n* = 15)
Male, *n* (%)	9 (60)
Age (years old)	66 ± 12
LAD (mm)	43 ± 8
LVEF (%)	60 ± 8
Hypertension, *n* (%)	7 (47)
Diabetes mellitus, *n* (%)	2 (13)
eGFR (ml/min per 1.73 m^2^)	66 ± 11
BNP, pg/ml	130 ± 101
CHADS2 score (point)	1.3 ± 1.0
Initial AF ablation, *n* (%)	10 (67)
The time from MRI acquisition to the ablation (days)	95 ± 60
Atrial wall thickness (mm)	2.2 ± 0.3

Note

Values are presented as the mean ± SD or *n* (%).

Abbreviations: AF, atrial fibrillation; BNP, B‐type natriuretic peptide; eGFR, estimated glomerular filtration rate; LAD, left atrial dimension; LVEF, left ventricular ejection fraction; MRI, magnetic resonance imaging.

### 3.2 Distribution of the NPAs and AWT

**FIGURE 3:**
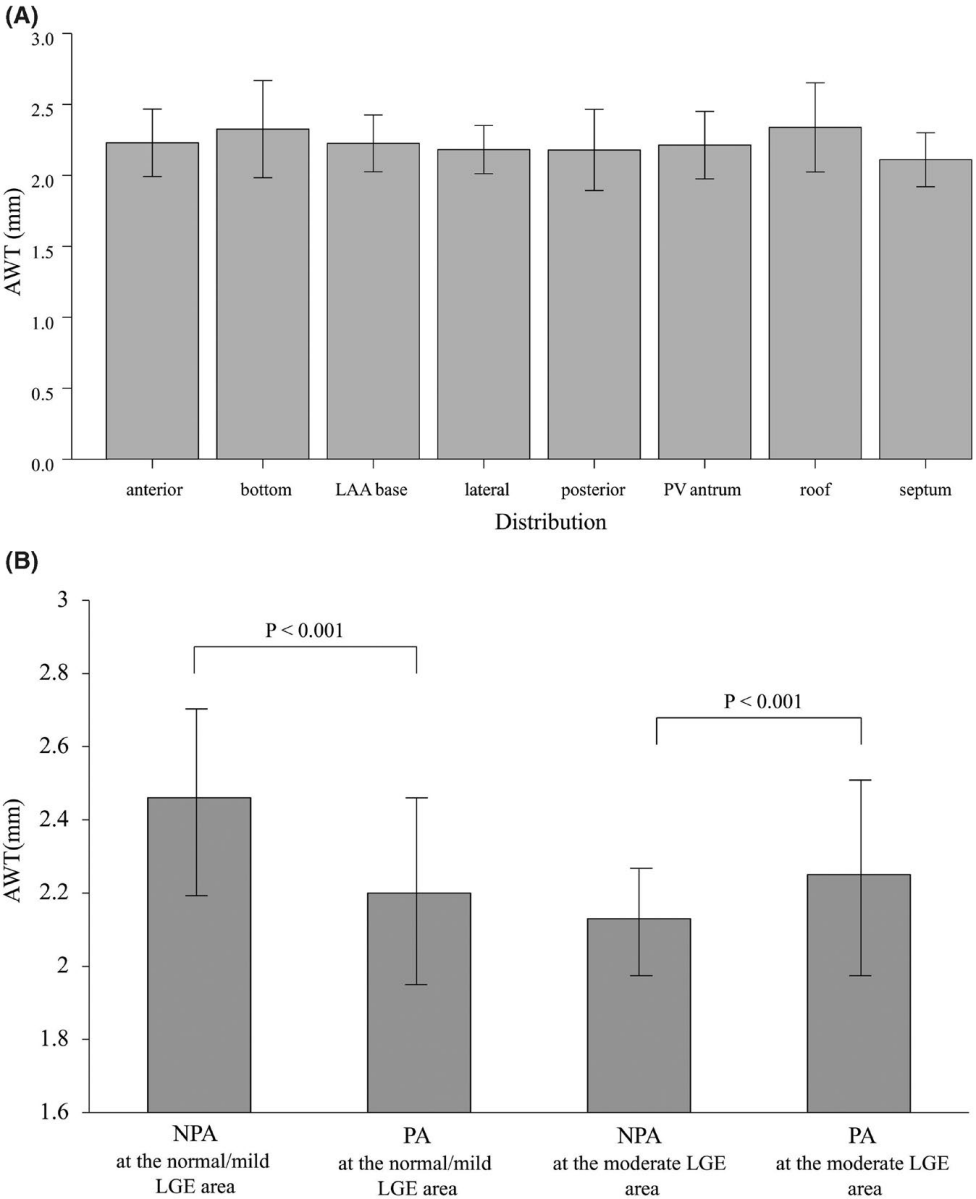
The mean AWT in each segment of the whole left atrium (A) and mean AWT in the NPAs and PAs in normal/mild LGE and all LGE areas (B). AWT, atrial wall thickness; LAA, left atrial appendage; LGE, late gadolinium enhancement; NPA, non‐passively activated area; PA, passively activated area. PV, pulmonary vein

NPAs were found in 61 (21%) of 287 segments. Although the NPAs were mostly found around the PV antrum (21 [34.4%] of 61 NPAs), the AWT did not differ at each segment (anterior: 2.3 ± 0.3 mm, bottom: 2.3 ± 0.2 mm, LAA base: 2.2 ± 0.2 mm, lateral: 2.2 ± 0.2 mm, posterior: 2.0 ± 0.4 mm, PV antrum: 2.3 ± 0.3 mm, roof: 2.4 ± 0.4 mm, and septum: 2.1 ± 0.2 mm, *p* = .094) (Figure3A).

### 3.3 Relationship between the AWT and LGE volume‐ratio

**FIGURE 4:**
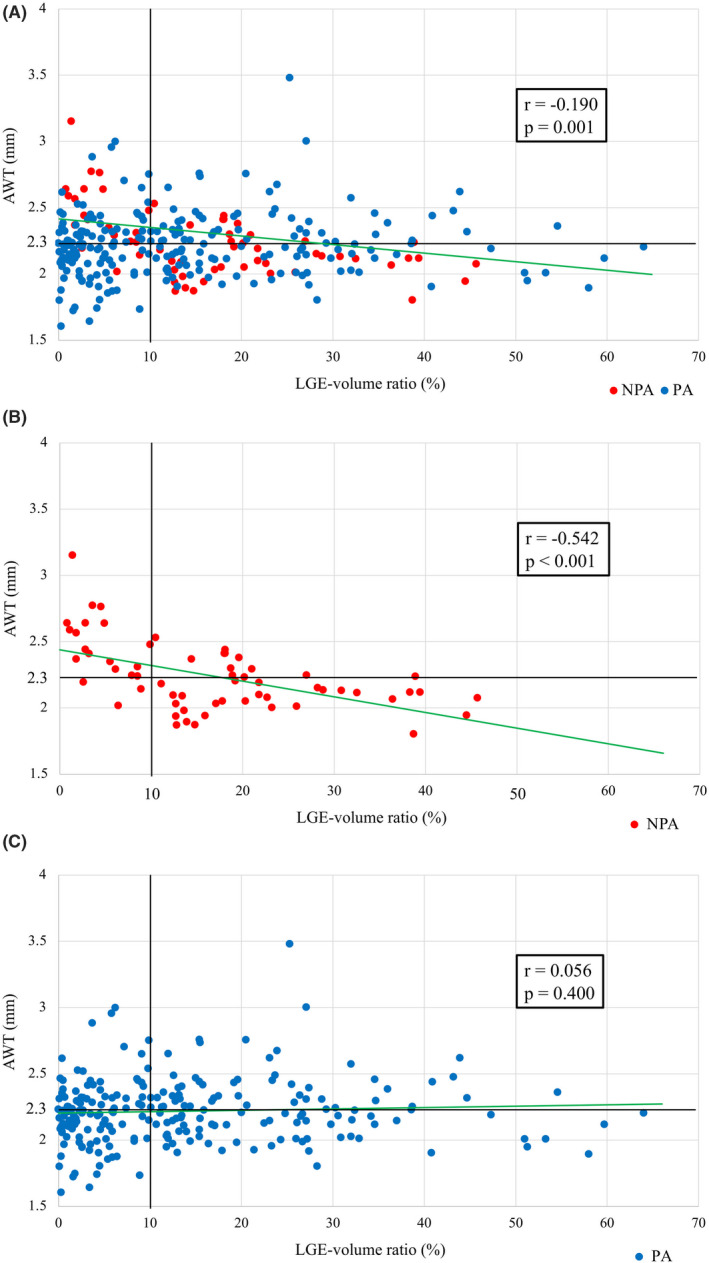
The distribution of the NPAs and PAs according to the AWT and LGE‐volume ratio. The NPAs (red) and PAs (blue). The AWT correlated negatively with the LGE‐volume ratio in the total areas (A). This correlation was stronger in the NPAs (B) than PAs (C). AWT, atrial wall thickness; LGE, late gadolinium enhancement; NPA, non‐passively activated area; PA, passively area

The distribution of the NPAs and passively activated areas (PAs) according to the AWT and LGE‐volume ratio are shown in Figure 4. Normal/mild LGE areas were found in 131 (45.6%) of 287 areas and moderate LGE areas in 156 (54.4%) of 287 areas. The AWT correlated negatively with the LGE‐volume ratio among the total areas (*r* = −.190, *p* = .001) (Figure 4A). Of note, this correlation was significant for the NPAs but not the PAs (NPA: *r* = −.542, *p* < .001; PA: *r* = .056, *p* = .400) (Figure 4B,C).

### 3.4 NPAs and the AWT in normal/mild LGE areas

NPAs were found in 20 (15.3%) of 131 normal/mild LGE areas where the AWT was significantly thicker in the NPAs than PAs (NPAs: 2.5 ± 0.3 mm vs. PAs: 2.2 ± 0.3 mm, *p* < .001). However, NPAs were found in 41 (26.3%) of 156 moderate LGE areas where the AWT was thinner than that of the PAs (NPAs: 2.1 ± 0.2 mm vs. PAs: 2.3 ± 0.3 mm, *p* < .01) (Figure 3B).

### 3.5 Optimal AWT of normal/mild LGE areas predicting AF rotors

**FIGURE 5:**
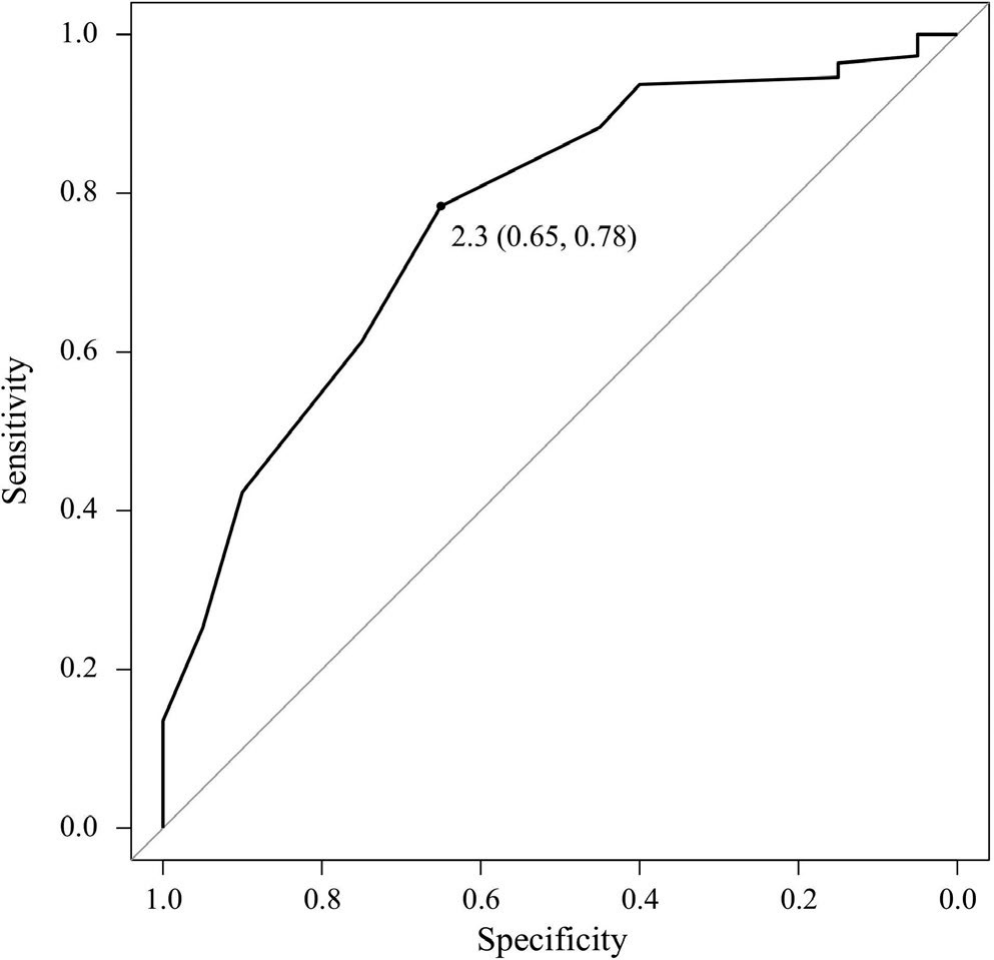
Optimal AWT of normal/mild LGE areas predicting AF rotor. An ROC curve analysis yielded an optimal cutoff value of 2.3 mm and the AUC was 0.77 (0.66–0.88) for predicting the presence of an NPA in normal/mild LGE areas. AF, atrial fibrillation; AWT, atrial wall thickness; LGE, late‐gadolinium enhancement

**FIGURE 6:**
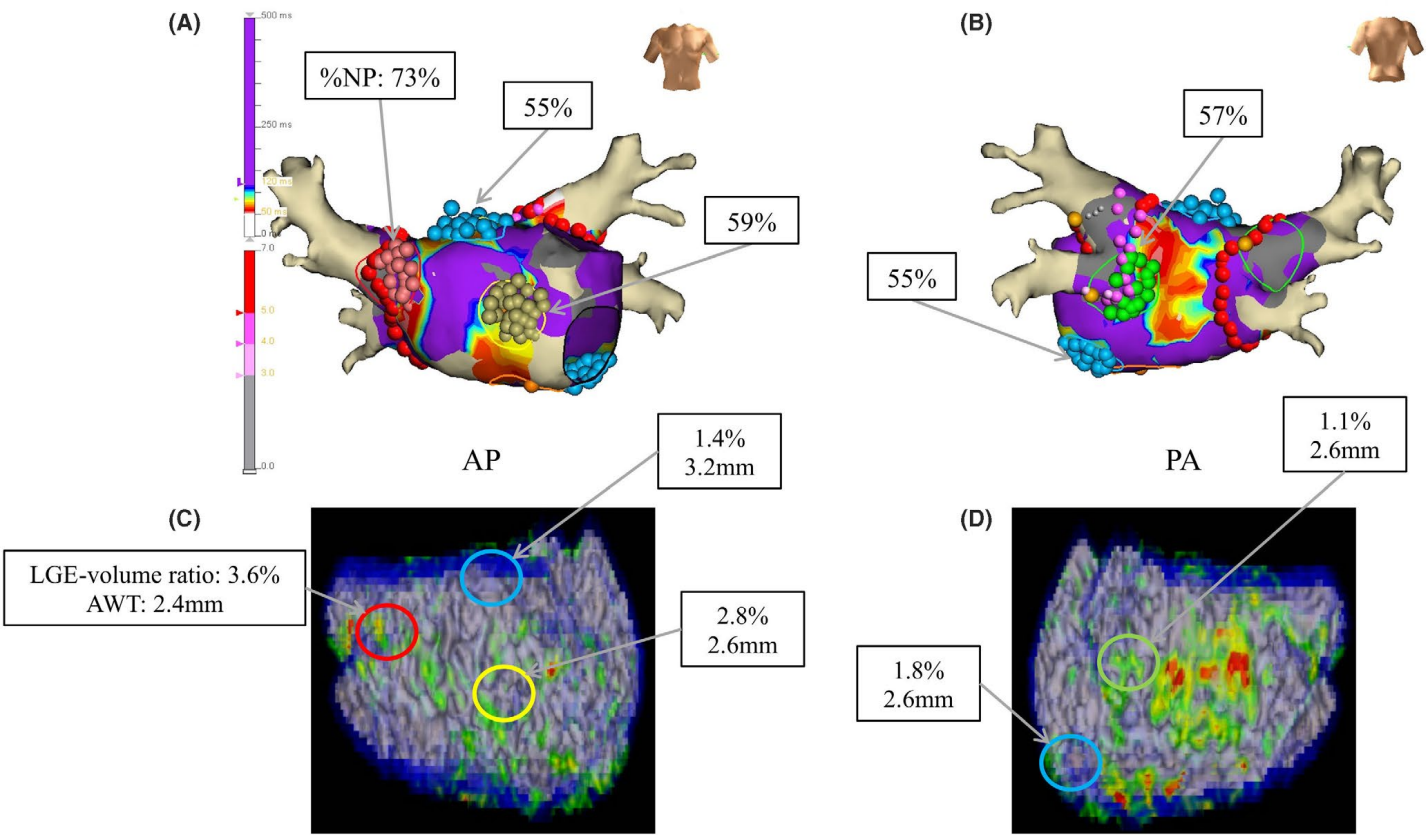
Representative case in our study. ExTRa Mapping with the NavX system in the AP (A) and PA (B) views. The red, yellow, green, and light blue circles indicate the NPAs with a high %NP of 73%, 59%, 57%, and 55%, respectively. The 3D LGE‐MRI of the LA in the AP (C) and PA (D) views. The red, yellow, green, and light blue circles correspond to those in panel (A). Despite the low value of the LGE‐volume ratio, these three areas were determined to be NPAs and their AWT was thick. %NP, non‐passively activated ratio; AP, anterior‐posterior; AWT, atrial wall thickness; LA, left atrium; LGE‐MRI, late‐gadolinium enhancement magnetic resonance imaging; LSI, lesion size index; NPA, non‐passively activated area; PA, posterior‐anterior

An ROC curve analysis yielded an optimal cutoff value of 2.3 mm and the AUC was 0.77 (0.66–0.88) for predicting the presence of an NPA in normal/mild LGE areas (Figure 5). As for the optimal AWT, the sensitivity, specificity, and positive and negative predictive values for the cutoff values were 65.0%, 78.4%, 35.1%, 92.6%, respectively. A representative case is shown in Figure 6. Five NPAs were found in normal/mild LGE areas where the AWT was thicker than 2.3 mm.

### 3.6 Comparison of the proportion of MRs/MWs in the %NP between normal/mild and moderate LGE areas

**FIGURE 7:**
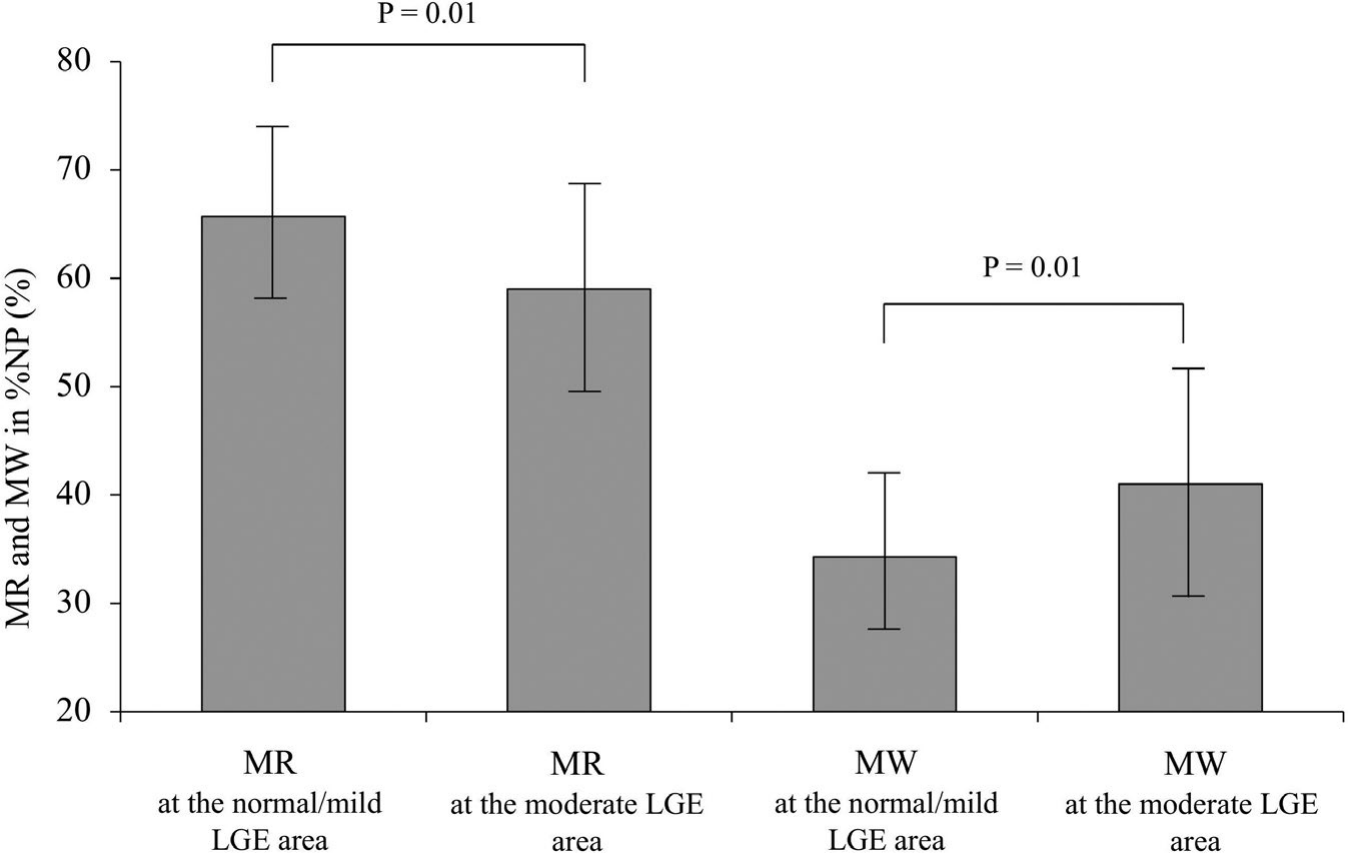
The proportion of MRs and MWs in the %NP of NPAs in normal/mild LGE and heterogenous LGE areas. %NP, non‐passively activated ratio; LGE, late gadolinium enhancement; MR, meandering rotors; MW, multiple wavelets; NPA, non‐passively activated area

The proportion of MRs in the %NP was significantly higher in normal/mild LGE areas than in moderate LGE areas (Normal/mild LGE: 65.7 ± 8% vs. Moderate LGE: 59.0 ± 10%, *p* = .01). On the other hand, the proportion of MWs in the %NP was significantly lower in normal/mild LGE areas than moderate LGE areas (Normal/mild LGE: 34.3 ± 8% vs. Moderate LGE: 41.0 ± 10%, *p* = .01) (Figure 7).

### 3.7 AWT at normal/mild LGE area and AF recurrence

AF recurrence was observed in 5 (33%) of 15 patients. Of them, two had NPAs at the normal/mild LGE areas where no direct RF application was attempted. In the 2nd procedure, NPAs were still found at the same region. The remaining three had NPA at the normal/mild LGE area around PV antrum, however no direct RF application was attempted, because it was included within PVI line. In the 2nd procedure, NPA recurred at the PV antrum owing to recondition of the PVI. Of interest, AWT was measured as 2.4, 2.6, and 2.7 mm, respectively.

## 4 DISCUSSION

### 4.1 Main findings

This study demonstrated that (1) the AWT correlated with the LGE‐volume ratio negatively in the NPAs, (2) the AWT in the NPAs was significantly thicker than that in the PAs in normal/mild LGE areas, and (3) the proportion of MRs in the %NP was significantly higher in normal/mild LGE areas than moderate LGE areas, whereas the proportion of MWs was higher in moderate LGE areas.

### 4.2 AWT on MRI images

Recently, computed tomography (CT) images have been used to estimate the AWT.^14^ Despite its high spatial resolution, CT is inherently low in soft‐tissue contrast, making the detection of atrial borders very difficult. The use of iodine‐based contrast agents can increase the contrast of the endocardial borders, however, identifying the epicardial borders remains challenging. Even if the fact that MRI is noninvasive and provides superior soft‐tissue contrast compared to CT, few MRI‐based studies have been reported to measure the AWT of the LA in AF patients. A previous study regarding the LA wall thickness measured by CT images demonstrated that the mean LA wall thickness in chronic AF and paroxysmal AF was 2.1 ± 0.2 mm and 2.4 ± 0.2 mm, respectively.^14^ In addition, Varela et al. presented MRI study to create the first whole atria atlas of wall thickness from images of 10 healthy volunteers. According to their results, the mean AWT was 2.4 ± 0.7 in LA. ^15^ In this study, the AWT measured by MRI was 2.2 ± 0.3 mm in 15 persistent AF patients, in good agreement with the previous study.

### 4.3 Correlation between the AWT and fibrosis

A recent study showed that a thicker LA wall was associated with a stronger atrial maintenance substrate in patients with LA enlargement assessed by echocardiography.^16^ An enlarged LA and a thickened LA wall might seem to be contradicted. A thickened LA wall may implicate the stage of inflammation, edema. With the progression of AF to end‐stage disease, remodeling may advance to atrial fibrosis, leading to a thinner AWT. Moreover, the observation of fractionated electrical activity with a low voltage on the electroanatomic map may be predictive of a high risk of AF initiation and persistence. The loss of an adaptive atrial thickening may be the tipping point at which fibrosis and scar become irreversible.^17^ In our study, the LA wall thickness was negatively correlated with the LGE‐volume ratio and the correlation was significant at only the NPAs but not the PAs, which was consistent with these previous results.

### 4.4 Impact of the AWT on AF rotors in normal/mild LGE areas

Recent computational studies of patient‐specific atrial models, based on the reconstruction of fibrosis from LGE‐MRI, have provided mechanistic insights into the role of fibrosis in the dynamics of electrical re‐entrant drivers sustaining AF. Zahid et al. have demonstrated that AF was sustained by re‐entrant drivers persisting in fibrosis border zones.^3^ We previously reported that the LGE properties in anchoring AF rotors predominantly consist of moderate LGE areas in persistent AF patients. However, AF rotors are also observed in normal/mild LGE areas.^7^ Therefore, we considered that there might be other structural factors related to the AF rotor besides fibrosis. Roy et al. reported that AWT gradients or fibrosis and both played an important role in anchoring AF rotors. Of important, they also reported that AF reentrant driver initiated from the area with AWT gradients in absence of fibrosis.^8^ In an optical mapping ex vivo study of perfused right atria from explanted diseased human hearts, activation delays between the endocardium and epicardium during atrial pacing were more prominent in areas with an increased wall thickness, transmural fiber orientation angle gradient, and interstitial fibrosis.^18^ Therefore, thicker parts of the LA could be the 3D rotational substrate perpetuating AF due to long activation delays between the endocardium and epicardium. However, those have not been validated in humans.

In our study, there was a significant difference in the proportion of MRs/MWs in the %NP between the normal/mild and moderate LGE areas. Handa et al. recently reported that the fibrosis pattern alters the mechanism of the fibrillatory organization and its persistence in Langendorff‐perfused rat hearts. They demonstrated that meandering rotational activation was mainly found with less fibrosis and less gap junction uncoupling and it disorganized into multiple wavelets in the progression of atrial fibrosis and gap junction uncoupling.^19^ Therefore, we speculated that the NPAs in normal/mild LGE areas might have been mainly caused by a complex fiber orientation in the three‐dimensionally large space between the endocardium and epicardium. This might facilitate long activation delays between the endocardium and epicardium, which result in a 3D rotational substrate perpetuating AF. To the best of our knowledge, this is the first human study focusing on the impact of the AWT in normal/mild LGE areas on AF rotors using LGE‐MRI.

### 4.5 NPAs with a greater AWT

A previous study provided simultaneous endo‐epicardial high‐density mapping data of breakthroughs during AF and demonstrated that the large majority of breakthroughs are explainable by transmural conduction.^20^ Recently, Parameswaran et al. analyzed simultaneously acquired endo‐epicardial right atrial recordings from 14 persistent AF patients undergoing cardiac surgery, collected with a high‐density grid electrode array (interelectrode distance of 3 mm).^21^ They demonstrated that endo‐epicardial dissociation is highly dynamic and wavefront propagation heterogeneous, suggesting that targeting a single focus of the endo‐epicardial dissociation or breakthrough is unlikely to prevent recurrence of AF. This is consistent with the concept of ExTRa Mapping, in which persistent AF encountered in clinical practice is mostly driven by spatially and temporally unstable rotors rather than stationary stable rotors.^5^ To rapidly predict the atrial excitation during AF, both a computer simulation (in silico) part and special artificial intelligence part were incorporated into the ExTRa Mapping system. The in silico part computed virtual atrial action potentials based on an in silico model of the human persistent AF in combination with the timing of the action potential generation determined by the intra‐atrial signals.^22^ Recent experiments have successfully shown that the phase map sequence of ExTRa Mapping is consistent with high‐resolution optical mapping.^6^ Ashihara et al. demonstrated a great catheter ablation outcome using ExTRa Mapping in persistent AF patients for maintaining sinus rhythm.^5^ This indicated that NPAs detected by ExTRa Mapping should contain the true AF rotors. Therefore, we believed that ExTRa Mapping would provide a more specific ablation target relevant to the cause of AF.

It was previously reported that AF recurrence correlated with the emergence of new AF rotors after catheter ablation, where they occurred in locations distinctly different from those of the original ones.^23^ To eliminate all of them, electrophysiological mapping, such as ExTRa mapping, should be performed repeatedly, however, it would result in a prolongation of the procedure time. Considering this issue, LGE‐MRI is useful for planning the ablation strategy, as it can narrow down the target to be ablated preoperatively.

### 4.6 Clinical implications

As we previously reported, the AF rotors were mainly be located in moderate LGE areas, which could be detected by LGE‐MRI.^7^ Preprocedural LGE‐MRI could evaluate the LA wall thickness as well as LGE areas precisely and would be useful to predict the AF rotors in the normal/mild LGE area. This would help in planning where to ablate in addition to the PVI at a point before the ablation procedure and might reduce frequent electrophysiological mapping. We believed that this would make a significant contribution to the realization of an AF ablation with a higher specificity. Finally, we strongly recommended that thinner AWT areas in normal/mild LGE areas should be excluded from the ablation targets.

### 4.7 Study limitations

Our study had several limitations. First, the sample size was relatively small. However, we focused on the association between the wall thickness and electrical properties of each segment. Fortunately, statistical significance could be found even in a total of 287 segments in 15 patients. Second, some patients underwent a prior ablation. In such cases, we could not completely discriminate between the ablation lesions and pre‐existing atrial fibrosis around the PVs. However, LGE was rarely observed on MRI before ablation. Furthermore, the LGE sites might have been overestimated on the posterior wall adjacent to the vertebrae and anterior wall adjacent to the aortic cusp because of wall compression by those organs. Moreover, it might have been difficult to measure the thickness of the posterior LA wall with consistency in all patients. Thirdly, the new phase‐mapping system adopted in this study may have had unknown limitations because it is widely used in Japan but not in other countries. We expect that this system will be widely used worldwide in the future. Fourth, mapping was not performed in the right atrium (RA) because of the stability of the mapping catheter and the reproducibility of the LGE‐MRI assessment in the RA. Additionally, as it is difficult to contact all electrodes of mapping catheter to LAA, mapping was not performed in the LAA. Fifth, considering the spatial resolution of our MRI, it was challenging to precisely measure the thin AWT. However, the wall thickness was only applied to the body of the LA and our results are consistent with that of the previous study. Furthermore, the inter‐ and intra‐ variability of measuring AWT were acceptable (inter‐observer: *r* = .87, *p* < .001; in intra‐observer: *r* = .94, *p* < .001). Finally, no histological validation was performed in the LGE areas. LGE‐MRI has a potential risk of over‐ and under‐estimating fibrosis.

## 5 CONCLUSIONS

The AF rotors were likely to be located in thick AWT areas in normal/mild LGE areas, which were possible ablation targets. Preprocedural LGE‐MRI was considered to be useful for identifying such specific areas associated with AF rotors.

## CONFLICT OF INTEREST

The Section of Arrhythmia is supported by an endowment from Medtronic Japan and Abbott Japan. Ken‐ichi Hirata chairs the Section, and Koji Fukuzawa and Kunihiko Kiuchi belong to the Section. However, all authors report no conflict of interest for this manuscript's contents.
